# UCP1 expression in human brown adipose tissue is inversely associated with cardiometabolic risk factors

**DOI:** 10.1093/ejendo/lvae074

**Published:** 2024-06-26

**Authors:** T’ng Choong Kwok, Lynne E Ramage, Alexandra Kelman, Karla J Suchacki, Calum Gray, Luke D Boyle, Scott I Semple, Tom MacGillivray, Gillian MacNaught, Dilip Patel, Edwin J R van Beek, Robert K Semple, Sonia J Wakelin, Roland H Stimson

**Affiliations:** University/BHF Centre for Cardiovascular Science, University of Edinburgh, Queen's Medical Research Institute, 47 Little France Crescent, Edinburgh EH16 4TJ, United Kingdom; University/BHF Centre for Cardiovascular Science, University of Edinburgh, Queen's Medical Research Institute, 47 Little France Crescent, Edinburgh EH16 4TJ, United Kingdom; University/BHF Centre for Cardiovascular Science, University of Edinburgh, Queen's Medical Research Institute, 47 Little France Crescent, Edinburgh EH16 4TJ, United Kingdom; University/BHF Centre for Cardiovascular Science, University of Edinburgh, Queen's Medical Research Institute, 47 Little France Crescent, Edinburgh EH16 4TJ, United Kingdom; Edinburgh Imaging Facility, Queen's Medical Research Institute, 47 Little France Crescent, Edinburgh EH16 4TJ, United Kingdom; University/BHF Centre for Cardiovascular Science, University of Edinburgh, Queen's Medical Research Institute, 47 Little France Crescent, Edinburgh EH16 4TJ, United Kingdom; University/BHF Centre for Cardiovascular Science, University of Edinburgh, Queen's Medical Research Institute, 47 Little France Crescent, Edinburgh EH16 4TJ, United Kingdom; Edinburgh Imaging Facility, Queen's Medical Research Institute, 47 Little France Crescent, Edinburgh EH16 4TJ, United Kingdom; Edinburgh Imaging Facility, Queen's Medical Research Institute, 47 Little France Crescent, Edinburgh EH16 4TJ, United Kingdom; Department of Radiology, Royal Infirmary of Edinburgh, 51 Little France Crescent, Edinburgh EH16 4SA, Scotland, United Kingdom; Department of Radiology, Royal Infirmary of Edinburgh, 51 Little France Crescent, Edinburgh EH16 4SA, Scotland, United Kingdom; University/BHF Centre for Cardiovascular Science, University of Edinburgh, Queen's Medical Research Institute, 47 Little France Crescent, Edinburgh EH16 4TJ, United Kingdom; Edinburgh Imaging Facility, Queen's Medical Research Institute, 47 Little France Crescent, Edinburgh EH16 4TJ, United Kingdom; University/BHF Centre for Cardiovascular Science, University of Edinburgh, Queen's Medical Research Institute, 47 Little France Crescent, Edinburgh EH16 4TJ, United Kingdom; Department of Surgery, Royal Infirmary of Edinburgh, 51 Little France Crescent, Edinburgh EH16 4SA, Scotland, United Kingdom; University/BHF Centre for Cardiovascular Science, University of Edinburgh, Queen's Medical Research Institute, 47 Little France Crescent, Edinburgh EH16 4TJ, United Kingdom

**Keywords:** obesity, aging, diabetes, cardiovascular disease, brown adipose tissue, thermogenesis, PET, UCP1

## Abstract

**Objective:**

Brown adipose tissue (BAT) is a therapeutic target for obesity. ^18^F-fluorodeoxyglucose positron emission tomography (^18^F-FDG PET) is commonly used to quantify human BAT mass and activity. Detectable ^18^F-FDG uptake by BAT is associated with reduced prevalence of cardiometabolic disease. However, ^18^F-FDG uptake may not always be a reliable marker of BAT thermogenesis, for example, insulin resistance may reduce glucose uptake. Uncoupling protein 1 (UCP1) is the key thermogenic protein in BAT. Therefore, we hypothesised that *UCP1* expression may be altered in individuals with cardiometabolic risk factors.

**Methods:**

We quantified *UCP1* expression as an alternative marker of thermogenic capacity in BAT and white adipose tissue (WAT) samples (*n* = 53) and in differentiated brown and white pre-adipocytes (*n* = 85).

**Results:**

*UCP1* expression in BAT, but not in WAT or brown/white differentiated pre-adipocytes, was reduced with increasing age, obesity, and adverse cardiometabolic risk factors such as fasting glucose, insulin, and blood pressure. However, *UCP1* expression in BAT was preserved in obese subjects of <40 years of age. To determine if BAT activity was also preserved *in vivo*, we undertook a case-control study, performing ^18^F-FDG scanning during mild cold exposure in young (mean age ∼22 years) normal weight and obese volunteers. ^18^F-FDG uptake by BAT and BAT volume were similar between groups, despite increased insulin resistance.

**Conclusion:**

^18^F-FDG uptake by BAT and *UCP1* expression are preserved in young obese adults. Older subjects retain precursor cells with the capacity to form new thermogenic adipocytes. These data highlight the therapeutic potential of BAT mass expansion and activation in obesity.

## Graphical Abstract

**Graphical Abstract:**
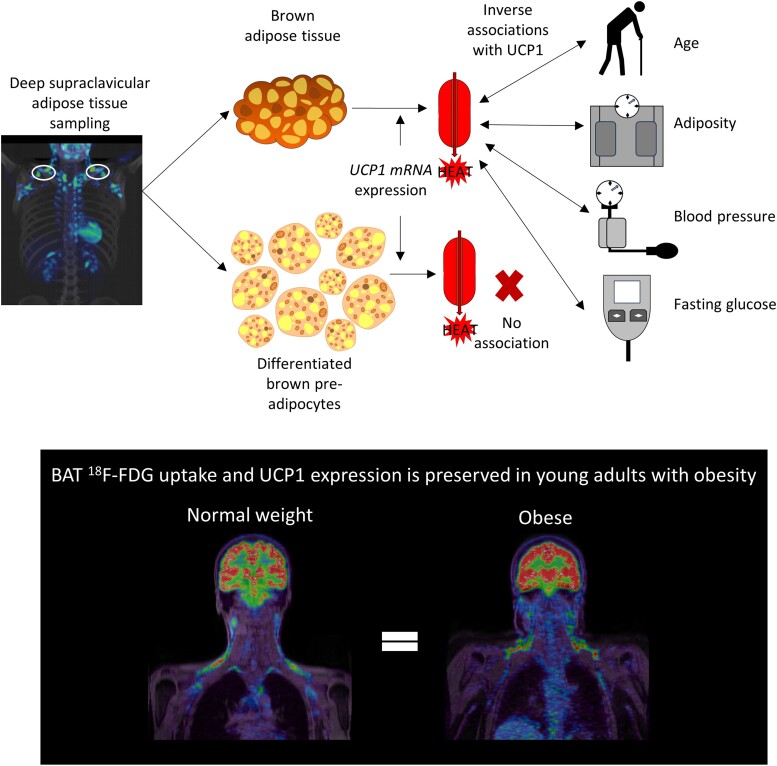

**Significance**

BAT activation offers therapeutic potential to treat obesity and cardiometabolic disease. Although detectable glucose uptake by BAT at room temperature is associated with improved cardiometabolic health, these findings may be confounded by obesity-induced insulin resistance and severely underestimates BAT prevalence. Here, we show that *UCP1* mRNA expression (the key thermogenic protein) in BAT (but not in differentiated brown pre-adipocytes) was inversely associated with aging, adiposity, hypertension, and insulin resistance, indicating defective BAT thermogenesis but the preservation of precursor cells in these groups of individuals. We also demonstrate that BAT *UCP1* expression and cold-induced glucose uptake by BAT is maintained in young adults with obesity despite substantial insulin resistance, highlighting the therapeutic potential of BAT activation in this group.

## Introduction

The prevalence of obesity increased substantially over the past 50 years, imposing significant population-wide morbidity and mortality.^1^ Obesity results from sustained net positive energy balance causing accumulation of adipose tissue.^1^ Adipose tissue is broadly divided into white (WAT) and brown (BAT) subtypes. WAT primarily subserves energy storage whereas BAT expends energy through non-shivering thermogenesis, most notably to maintain body temperature during cold exposure.^2^ To enable thermogenesis, brown adipocytes have numerous mitochondria, small lipid droplets, and rich sympathetic innervation.^3^ BAT thermogenesis relies primarily on a specialised thermogenic protein called uncoupling protein 1 (UCP1). UCP1 is located in the inner mitochondrial membrane of brown adipocytes and is activated with cold exposure via adrenergic stimulation, where it uncouples dissipation of the mitochondrial proton gradient from ATP production, creating an energy-generating futile cycle.^2^ There is significant interest in BAT as a potential pharmacological target in obesity and associated metabolic disease.^4^

BAT was thought to involute completely by adulthood, until the clinical use of ^18^F-fluorodeoxyglucose positron emission tomography (^18^F-FDG PET) imaging to diagnose malignancy led to the incidental discovery of activated BAT in adults.^5^ Adult BAT depots are typically located in the supraclavicular, paravertebral and occasionally perirenal regions, and biopsies from these depots reveal brown adipocytes that highly express UCP1.^5,6^ Early PET studies focused on BAT demonstrated reduced ^18^F-FDG uptake by BAT in older, obese, and diabetic people, highlighting its potential role in metabolic disease.^5,6^ However, aging and obesity are often interlinked and their relative effects on BAT are poorly understood.^3^

Clinical studies retrospectively analysing ^18^F-FDG PET scans performed at room temperature may underestimate BAT prevalence and function, given the importance of cooling for BAT activation.^5,7,8^ Although recent large scale ^18^F-FDG PET studies at room temperature have consistently demonstrated the protective effects of BAT with respect to diabetes, hypertension, dyslipidaemia, and coronary artery disease,^9,10^ it is unclear whether reduced BAT glucose uptake reflects a general reduction of BAT activity, particularly in older, obese people. For example, BAT activity as quantified using ^11^C-acetate was preserved in older subjects with type 2 diabetes despite reduced ^18^F-FDG uptake, implicating insulin resistance as a potential confounder of ^18^F-FDG PET.^11^ Reduced ^18^F-FDG uptake by BAT is also seen in individuals with genetic variations in *AKT2* known to cause insulin resistance,^12^ and in fasting-induced insulin resistance.^13^ Conversely, BAT mass may be reduced in older individuals irrespective of their diabetes status,^11^ while fatty acid uptake by BAT measured using ^18^F-fluoro-thiaheptadecanoic acid (^18^F-FTHA) was reduced in obesity.^14^ Therefore, it remains unclear whether total BAT mass and/or activity are reduced with age and/or obesity. Tissue *UCP1* expression serves as an independent marker of BAT thermogenic capacity. In this study, we measured *UCP1* mRNA levels in human supraclavicular BAT and WAT and assessed their relationship with cardiometabolic risk factors.

## Methods

All studies in this paper comply with the Declaration of Helsinki.

### BAT biopsy study protocol

Paired WAT and BAT samples were obtained by 2 neck surgeons from 138 patients undergoing elective thyroid or parathyroid surgery from May 2011 to January 2022 in the Royal Infirmary of Edinburgh (ethical approval numbers 10/S1102/39, 15/ES/0094, and 20/ES/0061). Adipose samples were collected from (1) the deep supraclavicular region, posterior to the lateral thyroid gland adjacent to either the oesophagus or longus colli muscle (designated BAT) and (2) from the superficial subcutaneous adipose tissue of the neck (designated WAT) as described previously.^15^ Local ethical approval and informed consent from each participant were obtained. *UCP1* mRNA content was quantified in whole adipose tissue (*n* = 53) and in differentiated pre-adipocytes (*n* = 85). Data collected included demographics, medical history, anthropometric measurements, and pre-operative blood test results (including full blood count and indices of renal, liver, and thyroid function within the preceding 3 months). Fat mass and percentage were measured using bioelectrical impedance analysis (Omron BF 302).

Fasting blood was obtained prior to anaesthetic induction and analysed for insulin by ELISA (Mercodia, Sweden), non-esterified fatty acids (NEFA) using a colorimetric assay (Wako, Germany), and for glucose, noradrenaline and lipid profile (all on an Abbot ARCHITECT ci4100 Integrated Analyser). Estimated glomerular filtration rate (eGFR) was calculated using the Chronic Kidney Disease Epidemiology Collaboration (CKD-EPI) equation.^16^ The Homeostatic Model Assessment for Insulin Resistance (HOMA-IR) index was calculated as described previously.^17^

### Primary human adipocyte culture

Cell culture was performed as previously described.^15^ In brief, the stromal vascular cell fraction was isolated from BAT and WAT before plating and culturing in Dulbecco's Modified Eagle Medium (DMEM) containing 10% foetal bovine serum (FBS). At 80% confluence, cells were differentiated in the above medium with the addition of 1 nM tri-iodothyronine, 20 nM insulin, 500 μM 3-isobutyl-1-methylxanthine, 500 nM dexamethasone, and 125 μM indomethacin for 7 days. Cells were cultured for a further 7 days in DMEM containing 10% FBS, 1 nM tri-iodothyronine, and 20 nM insulin, followed by RNA extraction and quantification of *UCP1* mRNA expression.

### RNA extraction and quantitative real time PCR

Whole adipose tissue samples were homogenised in Qiazol reagent using a TissueLyser (Qiagen, Crawley, UK). RNA was extracted from whole tissue and adipocytes using the RNeasy Lipid Kit (Qiagen) and reverse transcribed into cDNA generated using the Qiagen QuantiTect reverse transcription kit. qRT-PCR was performed using the Roche Lightcycler 480 with gene-specific primers (Invitrogen Ltd, Paisley, UK) and probes as previously described.^15^*UCP1* expression was expressed as the ratio of *UCP1* gene abundance to the abundance of control genes (*PPIA* and *RNA18S5*). The forward (F) and reverse (R) primer sequences (5′ to 3′) and corresponding Roche probe numbers were as follows: *PPIA* F: atgctggacccaacacaat R: tctttcactttgccaaacacc Probe number 48, *RNA18S5* F: cttccacaggaggcctacac R: cgcaaaatatgctggaacttt Probe number 46, and *UCP1* F: ctcaccgcagggaaagaa R: ggttgcccaatgaatactgc Probe number 25.

### ^18^F-FDG PET/MRI case-control study protocol

Ethical approval was obtained by the South East Scotland Research Ethics Committee (17/SS/0095, 20/SS/0024). All participants provided prior informed consent. Six normal weight and 6 age-matched obese but otherwise healthy volunteers were recruited. Inclusion criteria were as follows: Aged 18-35 years; body mass index (BMI) 18.5-25 kg/m^2^ (normal weight) or 30-45 kg/m^2^ (obese); weight change of less than 5% in the preceding 6 months; no acute or chronic medical conditions; taking no regular medications; no claustrophobia or other contraindication to undergoing a MRI scan; alcohol intake ≤ 14 units/week; screening blood tests within acceptable limits (full blood count, glucose, renal function, liver function, and thyroid function tests); not currently pregnant or lactating (female participants).

Volunteers attended the Edinburgh Clinical Research Facility in standard light clothing after overnight fast, after no exercise or alcohol for 2 days before the study visit. Anthropometric measurements (height, weight, waist and hip circumference, body fat percentage, blood pressure, and pulse) were recorded. Baseline blood samples were collected at room temperature for subsequent analysis of glucose (colorimetric kit [Sigma]), NEFAs, and insulin (analysed as above). Participants were placed in a room cooled to 16-17 °C for 2 h. After 1 h of cold exposure, participants received an intravenous injection of 75 MBq ^18^F-FDG and a PET/MR scan was performed 1 h later.

#### PET/MR image acquisition

PET/MR image acquisition was performed as previously described.^18^ Participants were placed supine on a Siemens mMR scanner (Siemens Healthineers GmbH, Erlangen, Germany). A MRAC_GRAPPA scan was acquired for each PET bed position to generate a standard umap for attenuation correction. Images were acquired at 1.34 and 2.56 ms (repetition time of 4.02 ms) using a 3D T1 weighted Dixon VIBE acquisition to generate a fat fraction map.

#### PET/MR image analysis

The images were analysed using Analyze (version 12.0, AnalyzeDirect). MR and PET images were registered. Fat fraction maps were generated using the following equation:


$$\begin{array}{rl} \mathrm{Fat}\;\mathrm{Fraction}(\mathrm{FF})(\text{\%})\;= & (\mathrm{Signal}\;\mathrm{intensities}\;(\mathrm{SI})\mathrm{of}\;\mathrm{Fat})/\\ & (\mathrm{SI}\;\mathrm{Fat}+\mathrm{SI}\;\mathrm{Water})\times 100. \end{array}$$

BAT depots were localised using the BARCIST protocol.^19^ Lean body mass (LBM) was calculated using the Janmahasatian equation, based on the participants’ total body weight (TBW), BMI, and gender, as detailed below^20^:


$$\mathrm{LB}M_{\mathrm{male}}=(9270\times \mathrm{TBW})/[6680+(216\times \mathrm{BMI})]$$

$$\mathrm{LB}M_{\mathrm{female}}=(9270\times \mathrm{TBW})/[8780+(244\times \mathrm{BMI})].$$

The threshold for ^18^F-FDG uptake to localise BAT in units of standardised uptake value (SUV) was corrected for LBM, using the following equation (SUV_threshold_ = 1.2/LBM).^19^ Voxels with a FF of <50% were removed to ensure analysis was confined to adipose tissue. A SUV_threshold_ was applied to the fused PET/MR images. Voxels meeting the FF and PET thresholds were analysed. Any ROIs within the brain were excluded manually. Images were eroded using a 3 × 3 × 1 voxel matrix to account for PET blooming and boundary artefacts. BAT volume, mean SUV, and total ^18^F-FDG uptake by BAT (BAT volume × mean SUV) were quantified.

### Statistical analysis

Data analysis was performed with SPSS Version 25 (IBM). Normality of data distributions were assessed using the Shapiro-Wilk's test. *UCP1* levels were not normally distributed so were logarithmically transformed to the base of 10. Data from the *UCP1* expression study are presented as mean ± SEM. Data from the ^18^F-FDG PET/MR study are presented as mean ± SD. The prevalence of high BAT *UCP1* levels between groups was compared by chi-square test. Differences between groups (normal weight vs obese and high vs low *UCP1*) were analysed by unpaired *t*-test and Mann-Whitney *U* test for normally and non-normally distributed data, respectively. Non-parametric paired data (*UCP1* expression in BAT vs WAT and brown vs white adipocytes) were analysed by Wilcoxon Signed Rank test. Correlation between variables were analysed with Pearson's and Spearman correlation for normally and non-normally distributed data, respectively. Predictors of high *UCP1* levels were determined using univariate binary logistic regression. Variables with a significant association on univariate regression were further investigated using multivariate binary regression analysis to determine if the association remained significant following correction for other variables. Differences in BAT volume and ^18^F-FDG uptake between normal weight and obese groups were analysed using ANCOVA to adjust for any effect of outdoor temperature on these measurements. *P* < .05 was considered significant.

## Results

### *UCP1* mRNA expression in neck adipose tissue

The study population ranged from 20 to 78 years of age, and the majority were female (85%). There were no differences in age, gender, adiposity, cardiometabolic risk factors, mean outdoor temperature in the week preceding surgery, nor in surgical procedures performed between the whole tissue and differentiated pre-adipocyte groups, except for greater diastolic blood pressure in the whole adipose tissue group (Table S1).

First, study participants were divided into tertiles based on BAT *UCP1* expression. However, minimal *UCP1* expression was noted in both the lower (median 0.002 arbitrary units [AU]) and middle tertiles (threshold > 0.01, median 0.06 AU), with only substantial *UCP1* expression found in the upper tertile (threshold > 2 AU, median 7.2 AU). Subjects from the lower and middle tertiles were also similar in terms of age, obesity, and cardiometabolic risk factors such as blood pressure, lipid, and glycaemic profile (Table S2). Therefore, the low and middle tertiles were grouped together and the study population was subsequently dichotomised into “high UCP1” (*n* = 17) and “low UCP1” (*n* = 36) groups, using a *UCP1* threshold of 2 AU.

**Figure 1.**
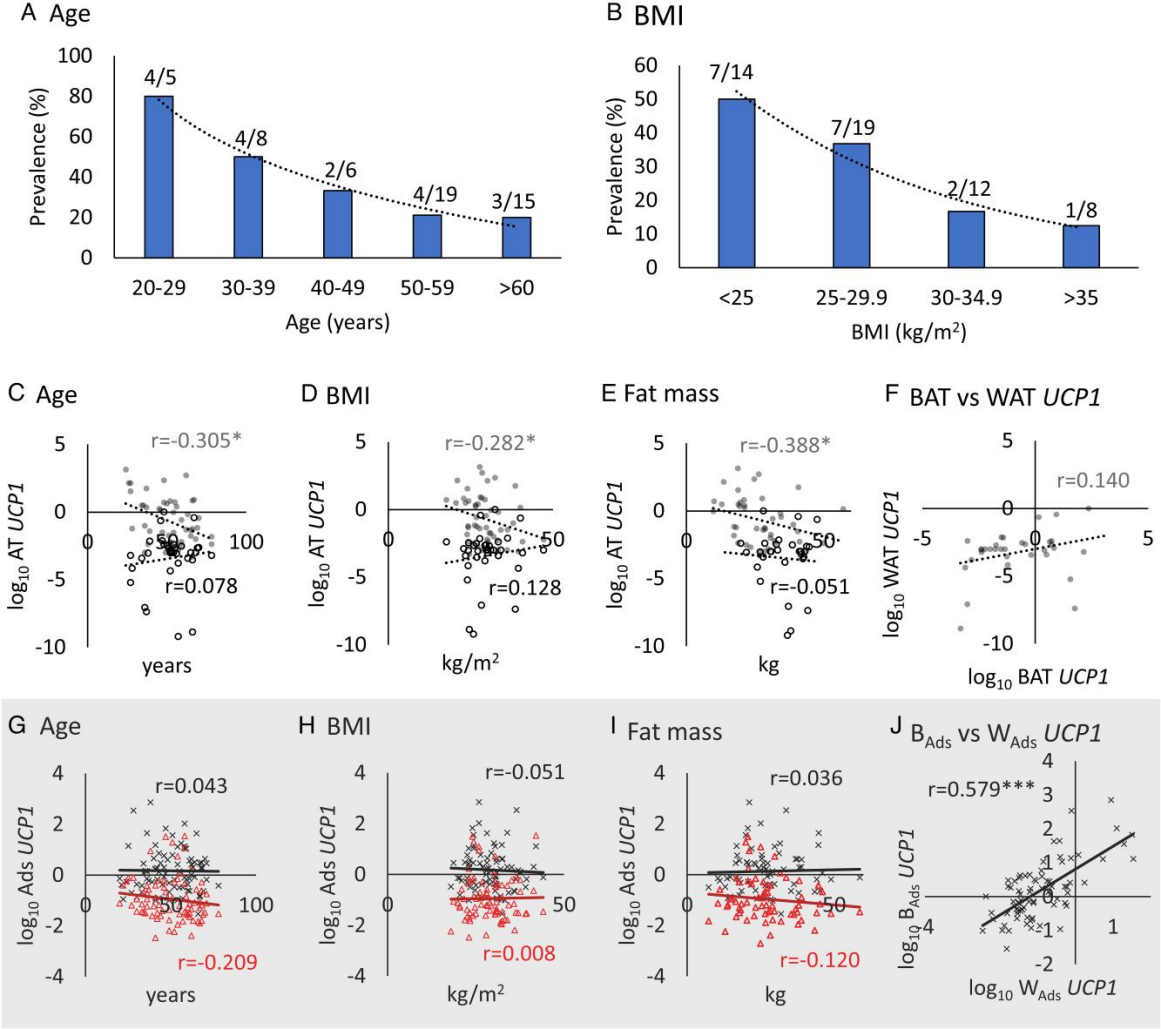
Association between adipose tissue and adipocyte UCP1 expression with age and adiposity. (A-F) UCP1 expression data in (A-F, clear panels) adipose tissue and (G-J, grey panels) adipocytes. (A and B) Data shown are the prevalence of UCP1 expression > 2 AU in human whole brown adipose tissue samples (*n* = 53) in patients divided into groups based on (A) age and (B) body mass index (BMI). The numbers above each column indicate the number of patients with UCP1 > 2 AU/total number participants within each age and BMI category. Data were analysed using the chi-square test for linear trend with the trendline depicted as a dotted line on each chart. (C-E) Correlation between UCP1 expression in BAT (filled circles) and WAT (open circles) with (C) age (*n* = 51 in BAT, 35 in WAT), (D) BMI (*n* = 51, 35), and (E) fat mass (*n* = 44, 29). (F) Correlation between BAT and WAT UCP1 (*n* = 36). (G-I) Correlation between UCP1 expression in brown (B_Ads_) (crosses) and white adipocytes (W_Ads_) (triangles) with (G) age (*n* = 84 in B_Ads_, 79 in W_Ads_), (H) BMI (*n* = 83, 78), and (I) fat mass (*n* = 75, 70). (J) Correlation between B_Ads_ and W_Ads_ UCP1 (*n* = 78). Correlation between normally and non-normally distributed data was analysed using Pearson’s and Spearman correlations, respectively. *P < .05, ***P < .001.

The high UCP1 group was younger, with lower BMI, waist and hip circumference, waist-hip ratio (WHR), waist-height ratio, fat mass, and fat percentage (Table 1). The proportion of people with high UCP1 progressively declined with increasing age and BMI (both *P* < .01) (Figure 1A and B). In the whole dataset, BAT *UCP1* levels correlated negatively with age (*r* = −0.305), weight (*r* = −0.374), BMI (*r* = −0.282), fat mass (*r* = −0.388), and waist circumference (*r* = −0.296, all *P* < .05), but not with WHR (*r* = −0.242, *P* = .11) (Figure 1C-E, Figure S1A).

**Table 1.** Anthropometric measurements, patient demographics, and biochemistry of individuals with high (*n* = 17) and low UCP1 levels (*n* = 36) in BAT.

	Low *UCP1* (<2 AU), *n* = 36	High BAT *UCP1* (≥2 AU), *n* = 17
Age (years)	55.7 ± 2.0	44.2 ± 3.5**
Gender (male/female, [% female])	8/28, [78%]	2/15, [88%]
Body weight (kg)	83.5 ± 3.0	71.8 ± 3.1*
Height (m)	1.66 ± 0.01	1.65 ± 0.02
BMI (kg/m^2^)	30.1 ± 1.0	26.5 ± 1.2*
Fat percentage (%)	35.3 ± 1.7	29.1 ± 2.6*
Fat mass (kg)	28.8 ± 1.7	20.9 ± 1.8*
Waist circumference (cm)	101.1 ± 2.6	87.9 ± 2.9*
Hip circumference (cm)	110.0 ± 1.9	101.9 ± 2.9*
Waist/hip ratio	0.92 ± 0.01	0.86 ± 0.01*
Waist/height ratio	0.61 ± 0.01	0.53 ± 0.02**
Systolic blood pressure (mmHg)	143 ± 4	131 ± 6*
Diastolic blood pressure (mmHg)	86 ± 2	79 ± 3*
Heart rate (beats per minute)	73 ± 2	81 ± 4
Outdoor temperature of preceding week (°C)	8.3 ± 2	7.7 ± 1.0
Fasting glucose (mmol/L)	5.5 ± 0.2	5.0 ± 0.1*
Insulin (mU/L)	10.7 ± 1.3	6.5 ± 0.8*
HOMA-IR	2.69 ± 0.35	1.44 ± 0.18*
NEFA (µM)	505 ± 45	544 ± 58
Total cholesterol (mmol/L)	5.2 ± 0.2	5.0 ± 0.3
HDL-C (mmol/L)	1.5 ± 0.1	1.4 ± 0.1
LDL-C (mmol/L)	3.5 ± 0.2	3.6 ± 0.3
Triglycerides (mmol/L)	1.2 ± 0.1	1.0 ± 0.2
Haemoglobin (g/L)	135 ± 4	136 ± 3
Haematocrit (L/L)	0.400 ± 0.012	0.400 ± 0.007
Mean cell volume (fL)	87 ± 3	88 ± 1
Platelet count (×10^9^/L)	251 ± 12	274 ± 19
White cell count (×10^9^/L)	6.7 ± 0.5	7.0 ± 0.7
Urea (mmol/L)	4.9 ± 0.5	5.2 ± 0.3
Sodium (mmol/L)	140 ± 1	140 ± 0
Potassium (mmol/L)	4.2 ± 0.2	4.3 ± 0.1
eGFR (mL/min/1.73 m^2^)	87 ± 3	101 ± 5*
TSH (mU/L)	1.3 ± 0.2	1.8 ± 0.4
T4 (pmol/L)	14 ± 1	12 ± 2
Bilirubin (µmol/L)	8 ± 1	14 ± 2***
Alanine transaminase (U/L)	21 ± 3	42 ± 9
Alkaline phosphatase (U/L)	100 ± 9	84 ± 12
Surgical intervention (thyroid/parathyroid/both)	12 (33%)/23 (64%)/1	10 (59%)/7 (41%)/0
Thyroid pathology (benign thyroid nodule/thyroid carcinoma/Graves’ disease)	5 (42%)/4 (33%)/3	4 (40%)/1 (10%)/5

Differences between groups were analysed by *t*-test and Mann-Whitney *U* test for normally and non-normally distributed data, respectively. Outdoor temperature measurements were based on temperature recordings obtained from the Edinburgh Airport weather station. *P < .05, **P < .01, ***P < .001 vs low UCP1.

Abbreviations: eGFR, estimated glomerular filtration rate; HDL-C, high-density lipoprotein cholesterol; HOMA-IR, Homeostatic Model Assessment for Insulin Resistance; LDL-C, low-density lipoprotein cholesterol; NEFA, non-esterified fatty acid; T4, thyroxine; TSH, thyroid stimulating hormone.

The high UCP1 group had lower fasting glucose and insulin concentrations, HOMA-IR, and systolic and diastolic blood pressure (Table 1). However, there were no correlations between any of these measures and BAT *UCP1* expression when analysed as continuous variables (Figure S1B-D). NEFAs, triglycerides, and cholesterol levels were similar between low and high UCP1 groups (Table 1).

BAT *UCP1* levels were lower in those with pre-existing hypertension (log *UCP1* −1.57 ± 0.27 vs −0.33 ± 0.32 AU, *P* < .01) (Figure S2A) and in those prescribed beta-blockers (log *UCP1* −2.22 ± 0.36 vs −0.57 ± 0.25 AU, *P* < .01) (Figure S2B). Gender and outdoor temperature did not alter the frequency of high UCP1 (Table 1). Serum bilirubin concentrations, while within the normal range in all participants, positively correlated with *UCP1* expression in BAT (*r* = 0.482, *P* < .05) (Table 1, Figure S1E). BAT *UCP1* expression also correlated positively with eGFR (*r* = 0.290, *P* < .05) (Table 1, Figure S1F), but did not correlate with markers of muscle mass such as LBM (*r* = −0.114), serum urea (*r* = −0.164), or creatinine concentrations (*r* = −0.198, all *P* > .05).

*UCP1* expression in WAT was ∼1500-fold lower (log *UCP1* −3.94 ± 0.39 vs −0.70 ± 0.24, *P* < .001) than in BAT, with corresponding mean cycle threshold (CT) values of 36.7 ± 0.4 in WAT and 30.7 ± 0.7 in BAT. There was no correlation between WAT and BAT *UCP1* expression (Figure 1F). Unlike in BAT, WAT *UCP1* expression was not associated with age, adiposity nor any other cardiometabolic risk factors (Figure 1C-E, Figure S1A-D).

### *UCP1* expression in brown and white adipocytes

*UCP1* expression was ∼13-fold higher in brown than in white adipocytes differentiated from stromovascular cells *ex vivo* (log *UCP1* 0.16 ± 0.09 vs −0.95 ± 0.09, *P* < .001), with mean CT values of 31.8 ± 0.3 in brown and 35.1 ± 0.3 in white adipocytes. Both cell types were subjected to the same differentiation protocol. *UCP1* expression was higher in adipocytes than in whole tissue in both depots, and *UCP1* expression in paired white and brown adipocytes was closely correlated (*r* = 0.579, *P* < .001) (Figure 1J). *UCP1* expression in brown or white adipocytes did not correlate with age, adiposity or other cardiometabolic risk factors (Figure 1G-I, Figure S1G-J).

### Effect of age on *UCP1* expression in BAT

**Figure 2.**
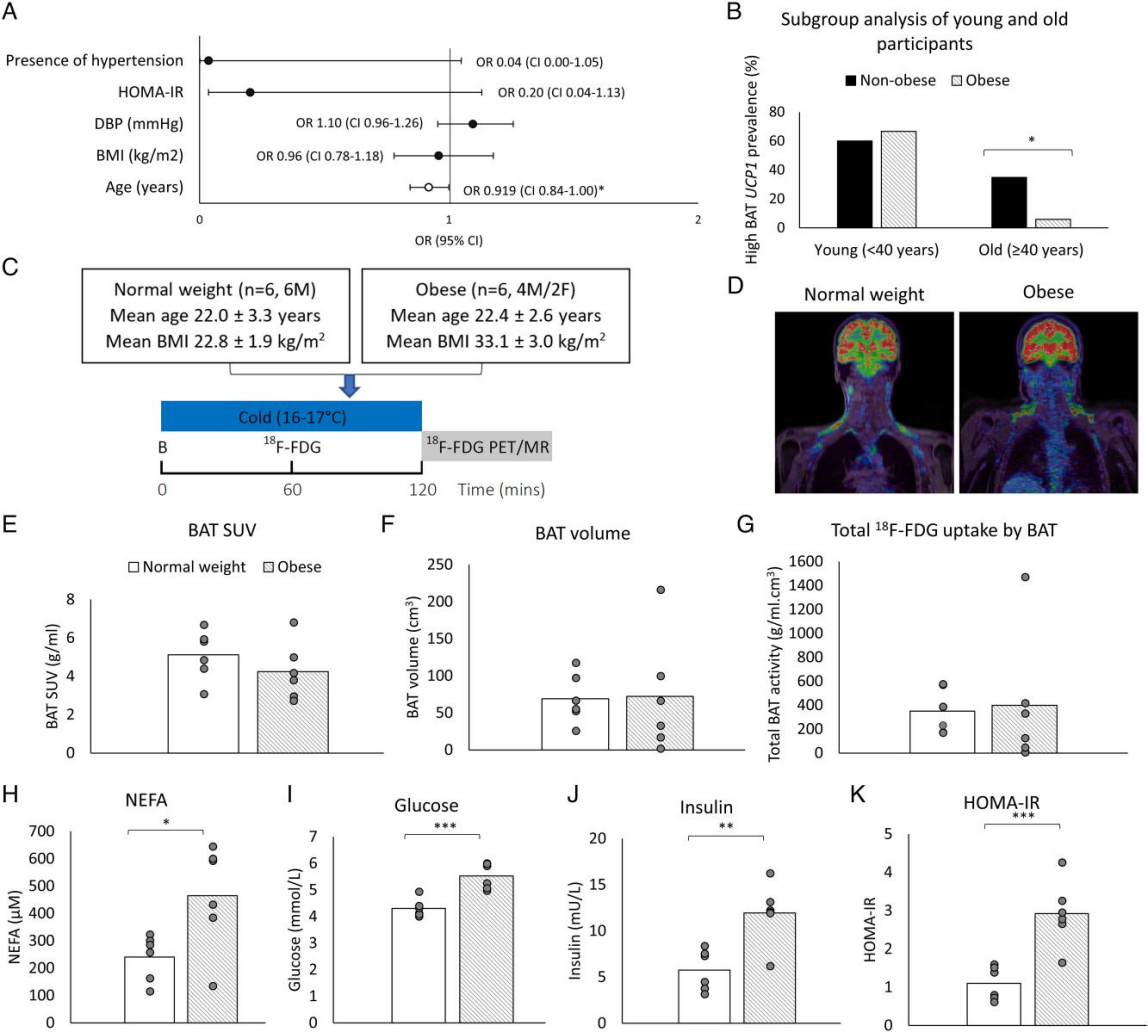
UCP1 expression and ^18^F-FDG uptake by BAT is maintained in young obese adults. (A) Multivariate regression analysis based on parameters with significant association from univariate analysis revealed age to be the primary predictor of high UCP1 expression in BAT (open circle). (B) Data are the percentage of BAT samples with high UCP1 levels in non-obese (black columns) and obese (striped columns) subjects in younger (<40 years) and older (≥40 years) subjects. (C) Protocol for *in vivo* case-control study testing whether ^18^F-FDG uptake by BAT was preserved in obesity in young adults following 2 h of mild cold exposure at 16 °C. Baseline blood samples (B) were obtained at room temperature. (D) Representative fused ^18^F-FDG PET/MR images in a normal weight and obese subject. (E-K) Data are presented as the mean with individual datapoints in grey circles for (E) ^18^F-FDG uptake by BAT using SUV, (F) BAT volume, and (G) total ^18^F-FDG uptake by BAT in normal weight (white columns) and obese subjects (striped columns, *n* = 6/group). (H-K) Data depict baseline fasting (H) NEFAs, (I) glucose, (J) insulin, and (K) HOMA-IR. Differences between categorical data were analysed by chi-square test, whereas differences between continuous data were analysed by *t*-test. Differences between ^18^F-FDG PET data were analysed using ANCOVA, adjusted for outdoor temperature. *P < 0.05, **P < 0.01, ***P < 0.001 obese vs normal weight participants. OR = odds ratio.

Next, we assessed which factors predicted high *UCP1* expression (>2 AU) in BAT using univariate regression. Predictors included younger age, lower adiposity, greater insulin sensitivity, lower diastolic blood pressure, and absence of hypertension (Table S3). We then undertook multivariate regression using BMI as the index of obesity. In this model, age was the only independent predictor of high *UCP1* in BAT (Figure 2A). Using other indices of adiposity, such as fat mass or WHR, in lieu of BMI did not alter this outcome. To investigate the relationship between aging and obesity further, we analysed BAT *UCP1* expression separately in younger (aged 18-39 years, *n* = 13) and older (aged ≥40 years, *n* = 40) participants. Obesity reduced the proportion of people with high *UCP1* expression only in the older group (Figure 2B). Conversely, mean BAT *UCP1* expression was lower in obese than in normal weight participants only in the older subgroup (log *UCP1* −1.58 ± 0.34 vs −0.61 ± 0.37, *P* < .05).

### ^18^F-FDG uptake by BAT in normal weight and obese young adults

The characteristics of the normal weight and obese healthy volunteers are detailed in Table S4, importantly subjects were age-matched. While outdoor temperatures during the study visits were ∼4 °C lower in the obese group, this was not significantly different (*P* = .33). ^18^F-FDG uptake by BAT was measured following 2 h of mild cold exposure (Figure 2C). Consistent with the BAT *UCP1* expression data presented above, ^18^F-FDG uptake by BAT (mean SUV 5.1 ± 1.3 [SD] vs 4.2 ± 1.5 g/mL, *P* = .28) and detectable BAT volume (69 ± 33 vs 72 ± 78 cm^3^, *P* = .81) were similar between normal weight and obese groups (Figure 2E-G), even after adjustment for outdoor temperature, although BAT FF (79.9 ± 1.0% vs 71.7 ± 0.6%, *P* < .001) was higher in the obese participants. Measurements of insulin resistance such as fasting glucose (5.5 ± 0.5 vs 4.3 ± 0.3 mmol/L, *P* = .0006), insulin (11.9 ± 3.3 vs 5.8 ± 2.2 mU/L, *P* = .0032), HOMA-IR (2.9 ± 0.8 vs 1.1 ± 0.4, *P* = .0009), and NEFAs (465 ± 191 vs 240 ± 83 µM, *P* = .0251) were substantially greater in the obese group (Figure 2H-K).

## Discussion

Our findings demonstrate that *UCP1* mRNA expression in human BAT is reduced with age, adiposity, hypertension, and insulin resistance, providing further evidence of a link between BAT and metabolic health in human adults. Individuals in the low UCP1 group also had larger waist circumference, waist-hip and waist-height ratios, suggesting that greater visceral adiposity is also associated with reduced UCP1 in BAT.^21,22^ We also show that *UCP1* expression in human neck WAT and differentiated brown or white adipocytes do not correlate with cardiometabolic risk factors. Our findings complement previous observations that the presence of detectable BAT at room temperature is associated with reduced prevalence of risk factors for cardiometabolic disease.^9^ Interestingly, *UCP1* expression in BAT was not associated with improved lipid profile, possibly reflecting a limited impact of BAT activation on circulating lipids in humans. This is consistent with evidence from acute and chronic administration of the beta3-agonist mirabegron, which did not improve total cholesterol, LDL-cholesterol, or triglyceride concentrations despite activating BAT, although chronic administration did increase HDL-cholesterol.^23-25^

Mechanisms whereby BAT might improve cardiometabolic health are unclear. The putative protective effects may be mediated directly by BAT thermogenesis, as suggested by ^18^F-FDG PET studies where those with detectable BAT have improved glucose homoeostasis following cold exposure.^26,27^ There is also emerging evidence that BAT is an endocrine organ, potentially exerting metabolic benefits indirectly through crosstalk with other organs, including WAT, skeletal muscle, and liver.^28^ It is also possible that these protective effects may be independent of BAT thermogenic capacity but instead reflect the sympathetic tone of the body, increasing energy expenditure and utilisation of triglyceride stores through NEFA uptake by other tissues such as skeletal muscles.^29,30^ However, the inverse relationship between blood pressure and BAT *UCP1* expression in our dataset argues against increased sympathetic activity as the sole explanation.^31^ However, these associations do not prove causation and the greater expression of *UCP1* in BAT may simply be a marker of improved cardiometabolic health.

The lack of any association between *UCP1* expression in differentiated brown adipocytes with age and adiposity suggest that brown pre-adipocytes are present even in older, obese subjects and that they retain the capacity for differentiation at least *in vitro*, highlighting the therapeutic potential of recruiting and activating BAT in these patients. These adipocytes were not stimulated for example with noradrenaline during cell culture, suggesting that there may be restraining factors *in vivo*, which limits the differentiation of pre-adipocytes in older obese individuals. The close correlation between *UCP1* expression of brown and white pre-adipocytes suggests that these factors may not be lineage-specific. These factors remain unknown but understanding these pathways driving BAT mass expansion (and possibly WAT browning) in older, obese subjects is vital to determine whether BAT mass can be expanded *in vivo* in this group. While it is possible that differences in differentiation could alter *UCP1* levels as UCP1 is only expressed in mature adipocytes, differences in differentiation were not the cause for variation in *UCP1* expression between cell types in our dataset, since brown adipocytes generally had lower levels of differentiation than white.

Extensive published data attest to the reduction of BAT mass and activity with aging. However, most studies have focused on older participants (mean age ∼40 years),^32,33^ while those including younger obese participants failed to age-match their groups stratified by adiposity.^3,34^ Therefore, the effects of obesity on BAT activity may have been confounded by age. Here, we demonstrated that BAT thermogenic capacity, quantified using *UCP1* expression as a surrogate measure, as well as BAT mass and activity (^18^F-FDG uptake) was preserved in young obese people, suggesting that age is an independent predictor of BAT function but more importantly highlighting the therapeutic potential of targeting BAT in this group, consistent with prior ^18^F-FDG PET data.^35,36^ Our ^18^F-FDG PET data also mirror the BAT *UCP1* expression findings, suggesting that ^18^F-FDG uptake is not substantially altered by insulin resistance at least in young adults, in contrast to existing data in older adults with and without T2DM.^11^ In addition, glucocorticoids acutely increased ^18^F-FDG uptake by BAT despite decreasing whole body glucose uptake, demonstrating that insulin resistance and reduced BAT glucose uptake do not always occur concurrently.^15^

Age was the only independent predictor of *UCP1* expression in BAT. The underlying mechanisms for age-related decline in BAT function remain unclear but may be secondary to impaired sympathetic drive, cellular senescence in the adipose niche, oxidative stress-induced mitochondrial dysfunction,^37,38^ or hormonal changes with aging.^39^ We speculate that obesity may accelerate the decline in *UCP1* expression with aging; possible explanations include through further blunting of sympathetic tone^18^ or increase in oxidative stress.^40^ While not significant due to broad confidence intervals, the odds ratio (OR) for both HOMA-IR and hypertension potentially demonstrated greater effects on BAT *UCP1* expression than age. It is possible with a larger sample size these or additional factors would independently predict *UCP* expression, while there may be other important drivers (eg, genetic variation) that predict differences between individuals.^41^ Our participants were primarily females, as the predominant gender undergoing thyroid and parathyroid surgery. Early ^18^F-FDG PET data performed at room temperature suggested increased prevalence of BAT in females,^5^ but PET scans performed following cold exposure have failed to demonstrate substantial differences between genders.^42,43^ In our study, BAT *UCP1* expression also did not differ between genders, supporting those data. These findings suggest that those gender differences are most likely due to BAT activation occurring at a higher room temperature in females, potentially due to the lower muscle mass in this group.

We also demonstrated novel associations between BAT *UCP1* expression with other biochemical parameters. Renal function correlated positively with BAT *UCP1* expression but it is unclear from our dataset if BAT has direct reno-protective effects as demonstrated in previous murine studies^44^ or if these findings are confounded by age and adiposity. There were no correlations between BAT *UCP1* expression with urea, creatinine, or LBM, indicating that the relationship observed with eGFR is unlikely to be due to differences in muscle mass. We also identified an association between BAT *UCP1* expression and bilirubin levels. Bilirubin induces WAT browning in mice, but its role in human BAT is unknown.^45^ In humans, individuals with mild hyperbilirubinaemia and Gilbert’s syndrome are protected against diabetes and cardiovascular diseases, suggesting that bilirubin has a positive impact on cardiometabolic health.^46,47^ However, future studies will need to assess if these cardiometabolic protective effects are mediated through BAT activation.

This study has several limitations. We only measured *UCP1* mRNA expression in adipose tissue samples, which may not correlate with UCP1 protein or *in vivo* BAT thermogenesis and there are no existing data detailing this relationship. The measurement of UCP1 protein and expression of other genes involved in BAT thermogenesis (including UCP1-independent mechanisms^48^) were not possible due to lack of remaining samples for analysis. Due to the sample size available for these analyses, it is likely that we were underpowered to detect the significance of several variables on univariate analysis, such as fasting glucose and triglyceride levels as predictors of high BAT *UCP1* expression, as evidenced by the broad confidence intervals on regression analysis and a non-significant *P*-value despite having an OR of <0.5. Future analysis of larger datasets will be required to determine the significance of some of these variables. Additionally, we did not perform ^15^O or ^11^C-acetate PET imaging in our human *in vivo* study. Although BAT activity quantified using ^18^F-FDG PET imaging can be reduced compared with oxidative metabolism measured using ^15^O or ^11^C-acetate in some situations,^11,49^ our data clearly demonstrate preservation of BAT activity and thermogenic capacity in young obese individuals. We were also unable to run non-inferiority analysis on our ^18^F-FDG PET study due to the small sample size. While the mean SUV and BAT volumes were very similar between normal weight and obese participants, it is possible that a greater sample size could reveal small differences between groups. Finally, we were unable to compare the direct relationship between whole tissue and differentiated pre-adipocyte *UCP1* expression in the same subjects. Although these samples were obtained from different participants, both groups had similar characteristics and were therefore comparable (Table S1).

## Conclusions

In conclusion, BAT *UCP1* mRNA expression is reduced in older, obese people with hypertension and impaired glucose homoeostasis, in keeping with defective BAT thermogenesis. However, these people retain brown pre-adipocytes with the capacity to form new thermogenic adipocytes with appropriate stimulation *ex vivo*, highlighting its therapeutic potential in all individuals irrespective of their age and adiposity. BAT activity and *UCP1* levels are preserved in young obese adults, suggesting that they could be a valid target group for therapeutic interventions to active BAT.

## Supplementary Material

lvae074_Supplementary_Data
