# Supplementary material for: UCP1 expression in human brown adipose tissue is inversely associated with cardiometabolic risk factors
Source: Eur J Endocrinol. 2024 Jun 26;191(1):106–15. doi: 10.1093/ejendo/lvae074 (PMC11265601; doi:10.1093/ejendo/lvae074)
Supplement: lvae074_Supplementary_Data [file lvae074_supplementary_data.zip › eje-23-0869-File008.docx]

***Table S1 Participant characteristics for UCP1 expression study***

*Data are mean ± SEM for subjects where UCP1 expression was quantified in whole adipose tissue (n=53) or in differentiated pre-adipocytes (n=85). Comparisons between groups were analysed using unpaired t-test and chi-square test for continuous and categorical data respectively. Outdoor temperature measurements were obtained from the Edinburgh Airport weather station. *P<0.05 between whole adipose tissue and differentiated pre-adipocyte groups. HDL-C = high-density lipoprotein cholesterol, HOMA-IR = Homeostatic Model Assessment for Insulin Resistance, LDL-C = low-density lipoprotein cholesterol.*

|  | **Whole adipose tissue (n=53)** | **Differentiated pre-adipocytes (n=85)** |
| --- | --- | --- |
| Age (years) | 52.0 ± 1.9 | 49.7 ± 1.5 |
| Gender (Males/Females, [% females]) | 10/43, [18.9%] | 11/74, [87.1%] |
| Body weight (kg) | 79.8 ± 2.3 | 79.8 ± 2.0 |
| Height (m) | 1.66 ± 0.01 | 1.66 ± 0.01 |
| BMI (kg/m^2^) | 29.0 ± 0.8 | 28.9 ± 0.6 |
| Fat percentage (%) | 33.4 ± 1.5 | 33.7 ± 1.0 |
| Fat mass (kg) | 26.4 ± 1.4 | 27.0 ± 1.2 |
| Waist circumference (cm) | 94.9 ± 2.7 | 97.0 ± 2.0 |
| Hip circumference (cm) | 105.0 ± 2.5 | 110.0 ± 2.8 |
| Waist/hip ratio | 0.90 ± 0.01 | 0.88 ± 0.01 |
| Systolic blood pressure (mmHg) | 139 ± 3 | 132 ± 2 |
| Diastolic blood pressure (mmHg) | 84 ± 2 | 80 ± 1* |
| Heart rate (beats per minute) | 76 ± 2 | 75 ± 2 |
| Fasting blood glucose (mmol/L) | 5.3 ± 0.1 | 5.3 ± 0.1 |
| Insulin (mU/L) | 9.4 ± 0.9 | 10.9 ± 0.8 |
| HOMA-IR | 2.31 ± 0.24 | 2.64 ± 0.22 |
| Total cholesterol (mmol/L) | 5.1 ± 0.12 | 5.2 ± 0.1 |
| HDL-C (mmol/L) | 1.5 ± 0.1 | 1.6 ± 0.1 |
| LDL-C (mmol/L) | 3.5 ± 0.1 | 3.4 ± 0.1 |
| Triglycerides (mmol/L) | 1.1 ± 0.1 | 1.3 ± 0.1 |
| Outdoor temperature of preceding week (°C) | 8.1 ± 0.6 | 8.8 ± 0.5 |
| Surgical intervention (Thyroid/Parathyroid/Both) | 22 (42%)/30 (57%)/1 | 37 (44%)/46 (54%)/2 (2%) |
| Thyroid pathology (Benign thyroid nodule/thyroid carcinoma/Graves’ disease) | 9 (41%)/5 (23%)/8 | 17 (46%)/10 (27%)/10 (27%) |
