# Supplementary material for: UCP1 expression in human brown adipose tissue is inversely associated with cardiometabolic risk factors
Source: Eur J Endocrinol. 2024 Jun 26;191(1):106–15. doi: 10.1093/ejendo/lvae074 (PMC11265601; doi:10.1093/ejendo/lvae074)
Supplement: lvae074_Supplementary_Data [file lvae074_supplementary_data.zip › eje-23-0869-File009.docx]

***Table S2 Cardiovascular risk profile and biochemistry of participants in the lower and middle BAT* UCP1 *tertiles.***

*Data are mean ± SEM for subjects in the lower and middle BAT UCP1 tertiles (n=18 for each group). Differences between groups were assessed using the unpaired t-test and chi square test for continuous and categorical data respectively. Outdoor temperature measurements were based on temperature recordings obtained from the Edinburgh Airport weather station. HDL-C = high-density lipoprotein cholesterol, HOMA-IR = Homeostatic Model Assessment for Insulin Resistance, LDL-C = low-density lipoprotein cholesterol, NEFA = non-esterified fatty acid, T4 = thyroxine, TSH = thyroid stimulating hormone.*

|  | **Lower tertile (≤0.001AU), n=18** | **Middle tertile (0.001-2AU), n=18** |
| --- | --- | --- |
| Age (years) | 56.0 ± 2.8 | 55.4 ± 3.3 |
| Gender (Males / Females, [% females]) | 3 / 15, [80%] | 4 / 14, [71%] |
| Body weight (kg) | 83.6 ± 4.8 | 83.5 ± 3.6 |
| Height (m) | 1.66 ± 0.01 | 1.67 ± 0.02 |
| BMI (kg/m^2^) | 30.4 ± 1.8 | 30.0 ± 1.1 |
| Fat percentage (%) | 35.7 ± 1.6 | 35.0 ± 3.0 |
| Fat mass (kg) | 28.7 ± 2.3 | 28.8 ± 2.7 |
| Waist circumference (cm) | 101.1 ± 4.3 | 101.1 ± 2.9 |
| Hip circumference (cm) | 110.4 ± 3.0 | 109.6 ± 2.3 |
| Waist/hip ratio | 0.91 ± 0.02 | 0.92 ± 0.02 |
| Systolic blood pressure (mmHg) | 140 ± 4 | 147 ± 6 |
| Diastolic blood pressure (mmHg) | 83 ± 3 | 89 ± 2 |
| Heart rate (beats per minute) | 70 ± 3 | 77 ± 4 |
| Mean outdoor temperature of preceding week (°C) | 8.0 ± 1 | 8.6 ± 1.1 |
| Fasting blood glucose (mmol/L) | 5.3 ± 0.2 | 5.6 ± 0.3 |
| Insulin (mU/L) | 10.4 ± 1.9 | 11.0 ± 1.8 |
| HOMA-IR | 2.52 ± 0.48 | 2.84 ± 0.52 |
| NEFA (µM) | 537 ± 74 | 479 ± 59 |
| Total cholesterol (mmol/L) | 5.4 ± 0.2 | 5.0 ± 0.2 |
| HDL-C (mmol/L) | 1.7 ± 0.1 | 1.4 ± 0.1 |
| LDL-C (mmol/L) | 3.6 ± 0.2 | 3.5 ± 0.3 |
| Triglycerides (mmol/L) | 1.2 ± 0.1 | 1.1 ± 0.1 |
| Haemoglobin (g/L) | 135 ± 8 | 135 ± 2 |
| Haematocrit (L/L) | 0.396 ± 0.003 | 0.399 ± 0.006 |
| Mean cell volume (fL) | 87 ± 5 | 87 ± 1 |
| Platelet count (x10^9^/L) | 247 ± 22 | 254 ± 11 |
| White cell count (x10^9^/L) | 6.9 ± 0.6 | 6.6 ± 0.7 |
| Urea (mmol/L) | 5.1 ± 0.4 | 5.3 ± 0.5 |
| Sodium (mmol/L) | 140 ± 1 | 140 ± 1 |
| Potassium (mmol/L) | 4.3 ± 0.1 | 4.3 ± 0.1 |
| eGFR (mL/min/1.73m^2^) | 89 ± 4 | 86 ± 5 |
| TSH (mU/L) | 1.0 ± 0.2 | 1.6 ± 0.3 |
| T4 (pmol/L) | 14 ± 1 | 14 ± 1 |
| Bilirubin (µmol/L) | 8 ± 1 | 9 ± 1 |
| Alanine transaminase (U/L) | 23 ± 5 | 18 ± 3 |
| Alkaline phosphatase (U/L) | 112 ± 15 | 88 ± 11 |
