# Supplementary material for: UCP1 expression in human brown adipose tissue is inversely associated with cardiometabolic risk factors
Source: Eur J Endocrinol. 2024 Jun 26;191(1):106–15. doi: 10.1093/ejendo/lvae074 (PMC11265601; doi:10.1093/ejendo/lvae074)
Supplement: lvae074_Supplementary_Data [file lvae074_supplementary_data.zip › eje-23-0869-File010.docx]

***Table S3 Univariate analysis to determine predictors of high BAT* UCP1 *expression.*** *Univariate binary regression analysis was performed to determine the potential predictors of high BAT* UCP1 *mRNA levels****.*** *Variables highlighted in bold were associated with lower BAT UCP1 levels (P<0.05). CI = confidence interval, HDL-C = high-density lipoprotein cholesterol, HOMA-IR = Homeostatic Model Assessment for Insulin Resistance, LDL-C = low-density lipoprotein cholesterol, NEFA = non-esterified fatty acid, OR = odds ratio. NS = P>0.10*

| **Variables** | **OR** | **CI** | **p value** |
| --- | --- | --- | --- |
| **Age** | **0.933** | **0.887-0.981** | **0.007** |
| Gender | 0.888 | 0.199-3.960 | NS |
| Smoking (cigarettes per day) | 0.992 | 0.881-1.118 | NS |
| Current smoker status | 1.275 | 0.317-5.127 | NS |
| Alcohol intake (units per week) | 0.985 | 0.894-1.084 | NS |
| Height (m) | 0.169 | 0-249.444 | NS |
| **Weight (kg)** | **0.950** | **0.908-0.994** | **0.027** |
| **BMI (kg/m^2^)** | **0.875** | **0.770-0.993** | **0.039** |
| **Waist circumference (cm)** | **0.934** | **0.887-0.983** | **0.009** |
| **Hip circumference (cm)** | **0.931** | **0.871-0.995** | **0.036** |
| **Waist/hip ratio** | **0.166** | **0.030-0.928** | **0.041** |
| **Fat percentage (%)** | **0.911** | **0.839-0.990** | **0.028** |
| **Fat mass (kg)** | **0.900** | **0.822-0.986** | **0.023** |
| Systolic blood pressure (mmHg) | 0.972 | 0.742-1.003 | 0.077 |
| **Diastolic blood pressure (mmHg)** | **0.940** | **0.885-0.998** | **0.043** |
| Pulse (beats per minute) | 1.04 | 0.996-1.087 | 0.078 |
| NEFA (µM) | 1.001 | 0.998-1.004 | NS |
| Insulin (mU/L) | 0.834 | 0.690-1.007 | NS |
| Glucose (mmol/L) | 0.219 | 0.047-1.014 | 0.052 |
| **HOMA-IR** | **0.439** | **0.196-0.981** | **0.045** |
| Total cholesterol (mmol/L) | 0.827 | 0.395-1.733 | NS |
| HDL-C (mmol/L) | 0.599 | 0.097-3.687 | NS |
| LDL-C (mmol/L) | 1.062 | 0.532-2.118 | NS |
| Triglycerides (mmol/L) | 0.413 | 0.093-1.826 | NS |
| Polypharmacy (on >4 medications) | 0.300 | 0.073-1.231 | 0.095 |
| **Diagnosis of hypertension** | **0.087** | **0.010-0.734** | **0.025** |
