# Supplementary material for: UCP1 expression in human brown adipose tissue is inversely associated with cardiometabolic risk factors
Source: Eur J Endocrinol. 2024 Jun 26;191(1):106–15. doi: 10.1093/ejendo/lvae074 (PMC11265601; doi:10.1093/ejendo/lvae074)
Supplement: lvae074_Supplementary_Data [file lvae074_supplementary_data.zip › eje-23-0869-File011.docx]

***Table S4 Participant characteristics for ^18^F-FDG PET/MR study.*** *Data are mean ± standard deviation. Differences between weight groups were assessed with unpaired t-test. Outdoor temperature measurements were obtained at 8am based on readings from the Edinburgh Airport weather station, United Kingdom. *P<0.05, ***P<0.001 normal weight versus obese.*

|  | **Normal weight (n=6)** | **Obese (n=6)** |
| --- | --- | --- |
| Age (years) | 22.0 ± 3.3 | 22.4 ± 2.6 |
| Gender | 6 male | 4 male, 2 female |
| BMI (kg/m^2^) | 22.8 ± 1.9 | 33.1 ± 3.0*** |
| Fat percentage (%) | 14.8 ± 4.2 | 30.3 ± 6.4*** |
| Fat mass (kg) | 10.9 ± 3.3 | 30.9 ± 6.6*** |
| Waist circumference (cm) | 82.0 ± 4.8 | 107.4 ± 8.2*** |
| Hip circumference (cm) | 100.3 ± 3.7 | 115.7 ± 10.3*** |
| Waist/hip ratio | 0.82 ± 0.04 | 0.93 ± 0.07* |
| Baseline systolic blood pressure (mmHg) | 130 ± 12 | 129 ± 11 |
| Baseline diastolic blood pressure (mmHg) | 72 ± 11 | 83 ± 9 |
| Baseline heart rate (beats per minute) | 62 ± 6 | 77 ± 13* |
| Thermoneutral room temperature (°C) | 24.3 ± 1.2 | 24.0 ± 0.6 |
| Cold room temperature (°C) | 16.1 ± 0.8 | 16.9 ± 1.0 |
| Outdoor temperature (°C) | 8 ± 8.4 | 3.5 ± 4.7 |
