# Supplementary figures and images for: UCP1 expression in human brown adipose tissue is inversely associated with cardiometabolic risk factors
Source: Eur J Endocrinol. 2024 Jun 26;191(1):106–15. doi: 10.1093/ejendo/lvae074 (PMC11265601; doi:10.1093/ejendo/lvae074)

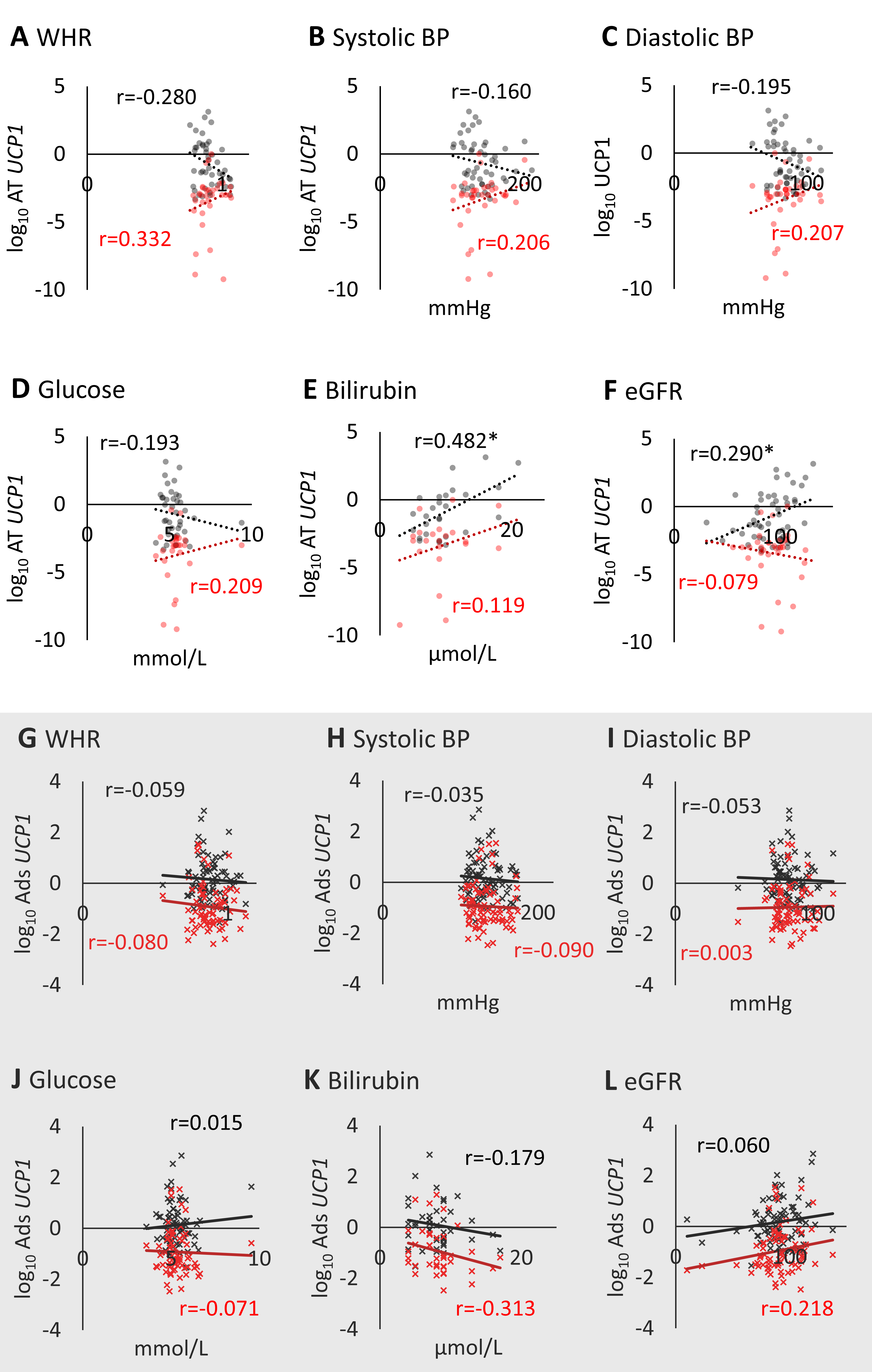

Supplement: lvae074_Supplementary_Data [file lvae074_supplementary_data.zip › eje-23-0869-File006.tif]

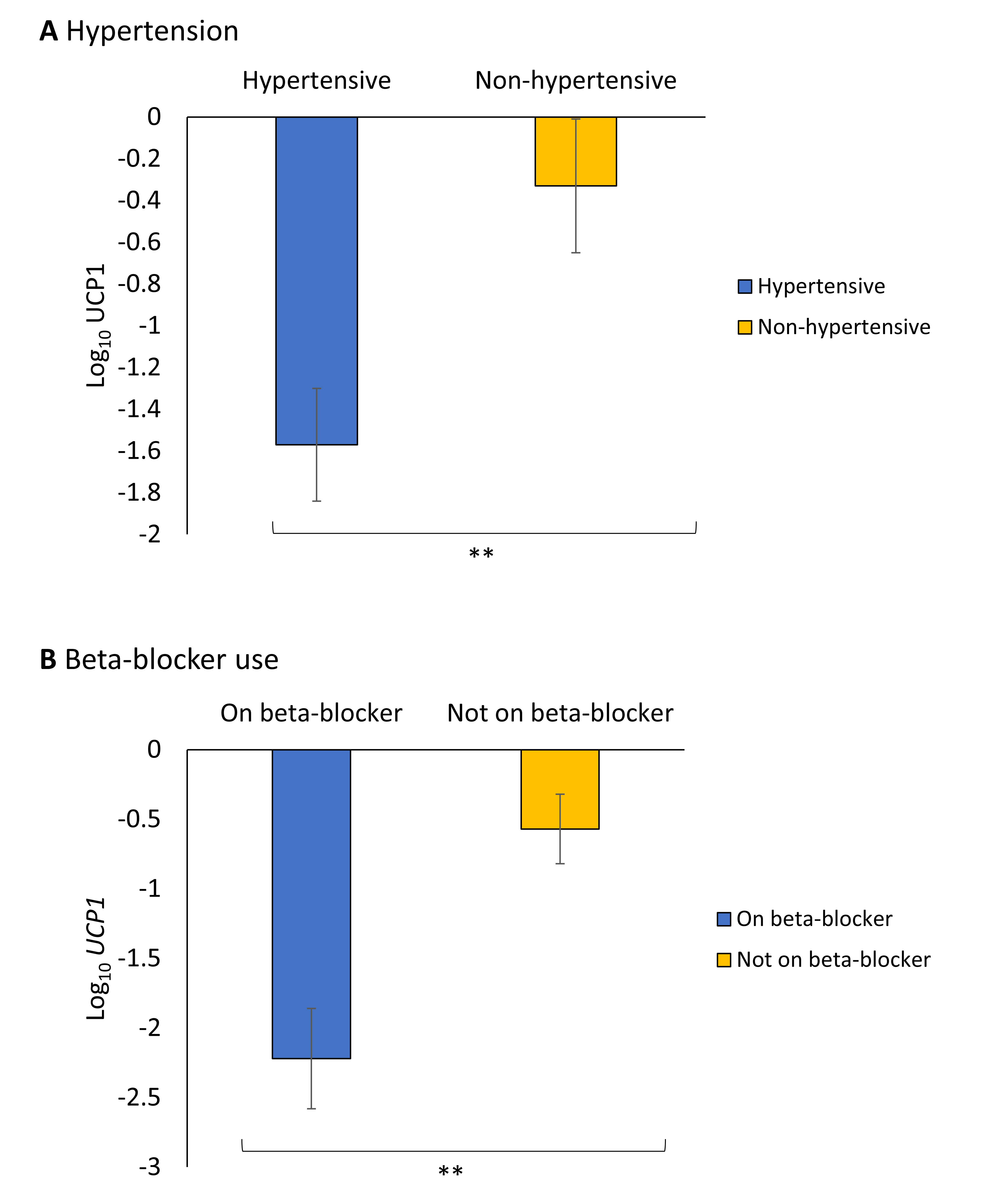

Supplement: lvae074_Supplementary_Data [file lvae074_supplementary_data.zip › eje-23-0869-File007.tif]
